# Object Vision in a Structured World

**DOI:** 10.1016/j.tics.2019.04.013

**Published:** 2019-05-27

**Authors:** Daniel Kaiser, Genevieve L. Quek, Radoslaw M. Cichy, Marius V. Peelen

**Affiliations:** 1 Department of Education and Psychology, Freie Universität Berlin, Berlin, Germany; 2 Donders Institute for Brain, Cognition and Behaviour, Radboud University Nijmegen, Nijmegen, The Netherlands; 3 Berlin School of Mind and Brain, Humboldt-Universität Berlin, Berlin, Germany; 4 Bernstein Center for Computational Neuroscience Berlin, Berlin, Germany

## Abstract

In natural vision, objects appear at typical locations, both with respect to visual space (e.g., an airplane in the upper part of a scene) and other objects (e.g., a lamp above a table). Recent studies have shown that object vision is strongly adapted to such positional regularities. In this review we synthesize these developments, highlighting that adaptations to positional regularities facilitate object detection and recognition, and sharpen the representations of objects in visual cortex. These effects are pervasive across various types of high-level content. We posit that adaptations to real-world structure collectively support optimal usage of limited cortical processing resources. Taking positional regularities into account will thus be essential for understanding efficient object vision in the real world.

## Positional Regularities in Object Vision

Many natural behaviors crucially depend on accurately perceiving objects in the environment. Consequently, understanding object vision has been a core endeavor in cognitive neuroscience for many years, and recent decades have yielded exciting insights into how the human visual system processes various types of objects [1–5]. By and large, these insights have come from studies investigating the processing of individual objects presented at arbitrary locations (usually at fixation). However, in natural vision many objects often appear in specific locations both with respect to visual space (e.g., airplanes in the sky) and relative to other objects (e.g., lamps above tables).

Although it has already been well established that such real-world positional regularities furnish observers with cognitive strategies that support effective behaviors (e.g., by providing schemata for economical memory storage [6–8] and efficient attentional allocation during search [9–11]), more recent work has begun to investigate the influence of real-world structure on how we perceive and represent objects. A rapidly burgeoning literature now indicates that positional regularities affect basic perceptual analysis both in terms of neural responses in visual cortex (e.g., by shaping tuning properties of object-selective regions) and perceptual sensitivity in psychophysical tasks (e.g., by facilitating object recognition and detection). Intriguingly, the general relevance of these effects has now been demonstrated across a range of high-level visual domains, including everyday objects, faces and bodies, words, and even social interactions between people. Drawing from both the neuroimaging and behavioral literatures, in this review we synthesize recent findings across processing levels and visual domains, and discuss how their resulting insights improve our understanding of real-world object vision.

## Adaptations to Absolute Locations in Individual-Object Processing

**Figure 1:**
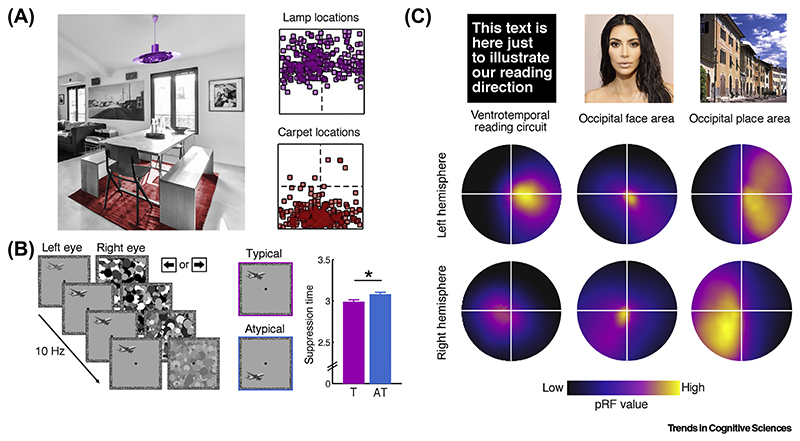
Neural Adaptations to Typical Absolute Object Locations. (A) The structure of natural scenes yields statistical regularities in the absolute positions of objects across visual space. Consequently, some objects tend to occupy particular visual field locations: unless directly fixated, lamps and carpets commonly appear in the upper and lower visual field, respectively. Scatter plots illustrate their position across 250 photographs from the LabelMe toolbox [129]. (B) In continuous flash suppression (CFS) experiments, the same objects gain preferential access to awareness (i.e., are detected faster) when they are typically positioned. Notably, because participants only needed to localize the target (no explicit recognition was required), the results indicate that typical absolute locations facilitate basic perceptual processing. Data reproduced from both experiments in [18]. Abbreviations: AT, atypical; T, typical. (C) Enhanced processing of typically positioned objects may be mediated by spatial neural tuning. Such tuning becomes apparent in the receptive field (RF) organization of category-selective regions, as uncovered by population RF (pRF) mapping studies. Even when measured with meaningless checkerboard stimuli, the coverage of visual space by the regions is consistent with spatial sampling of their preferred high-level contents: word-selective regions show a bias towards central vision and the horizontal meridian, face-selective regions have RFs close to the center of gaze, and scene-selective regions extensively cover peripheral space. Similarly, neurons coding individual objects may have RFs that preferentially cover areas of visual space in which these objects typically appear. Data reproduced from [24,31]. pRF maps cover 30° (words) or 20° (faces/scenes) of visual space; the color range has been adjusted to the maximum value of each region.

In natural environments, many objects appear at specific locations within a scene. For example, in indoor scenes, lamps are commonly found on the ceiling, whereas carpets are found on the floor. In natural vision these typical locations within a scene (in world-centered coordinates) translate to typical absolute locations within the visual field (in retinotopic coordinates). As a consequence, as we sample visual information from the scene, many objects - until directly fixated - are projected to specific locations in the visual field. Owing to their typical within-scene locations, for example, lamps tend to occur in the upper visual field and carpets tend to occur in the lower visual field (Figure 1A).

Recent studies have shown that these typical absolute locations in the visual field directly influence object perception: that is, the brain processes the same object differently depending on whether it appears at its typical visual field location or elsewhere. A recent fMRI study [12] used **multivariate pattern analysis** (MVPA; see Glossary) to decode the neural representations of individual objects (e.g., lamp or carpet) presented in either their typical or atypical visual field locations. Within object-selective lateral occipital cortex (LOC), decoding was more accurate when objects appeared at their typical location (e.g., a lamp in the upper visual field) than when these same objects appeared at an atypical location (e.g., a lamp in the lower visual field). This finding suggests that regularities in the absolute location of an object affect how it is encoded in the visual system, with sharper and more discriminable representations at retinal locations that correspond to its typical location in space.

Such effects are not confined to everyday objects but also extend to other stimulus classes: in occipitotemporal cortex, individual face and body parts evoke more distinct response patterns when they appear in their typical visual field locations (e.g., an eye in the upper visual field) compared to atypical locations (e.g., an eye in the lower visual field) [13,14]. Notably, these and other studies [15] have reported behavioral recognition advantages when faces, face parts, and body parts are shown in their typical locations, suggesting that adherence to real-world spatial structure facilitates both cortical processing and perceptual performance.

Performance benefits for typically positioned objects are even observable in simple detection tasks. In **continuous flash suppression** (CFS), interocular suppression renders a visual stimulus invisible for several seconds before it can be detected, and the time until it becomes visible (i.e., ‘breaks’ suppression) is considered to be a sensitive measure of the detectability of an object [16,17]. In such CFS designs, objects [18] and face parts [19] break suppression faster when presented in their typical absolute locations compared to atypical locations (Figure 1B). These effects are observed even when object identity is irrelevant for the task of the participant, suggesting that basic perceptual sensitivity for high-level stimuli is increased at their typical real-world locations.

That the typical absolute location of an object can impact upon its basic perceptual processing prompts the interpretation that these effects reflect changes in neural tuning properties. Evidence supporting this interpretation comes from electrophysiological studies which show that object representations are modulated by the position of the object in the visual field very soon following stimulus onset. Within the first 140 ms of vision, representations of both objects [20] and face parts [21] are strongest when the stimuli appear in their typical absolute locations, suggesting that location biases reflect neural tuning during perceptual stimulus analysis rather than solely post-perceptual feedback.

If the effects of typical positioning do not reflect post-perceptual feedback, how are they implemented within the visual architecture? One possible explanation comes from research exploring the **receptive field** (RF) organization of category-selective regions of occipitotemporal cortex. Studies using **population receptive-field mapping** [22,23] have revealed a startling functional correspondence between RF organization and category selectivity across high-level vision, showing that the RF properties of different category-selective regions are biased towards those parts of the visual field that are typically occupied by the preferred categories of the regions (Figure 1C).

For instance, RFs in word-selective cortex of English speakers are comparably small, biased towards foveal vision, and extend further horizontally than they do vertically [24,25]. This RF architecture mirrors the spatial sampling of written text during reading, which strongly relies on foveating and where information unfolds along the horizontal dimension. A complementary study [26] observed stronger responses to letters (but not to false fonts) along the horizontal meridian, corroborating the notion that word-specific activations are shaped by the direction of processing during reading.

A similar link between RF position and content-specific visual field biases is found in face- and place-selective cortices: face-selective regions have small RFs close to the center of gaze, consistent with the foveal processing necessary for individuating faces [27–31]. By contrast, place-selective regions have larger RFs that extensively cover peripheral visual space, consistent with the coarser spatial processing of natural scenes [29–32].

Taken together, these studies suggest that RF properties of high-level visual cortex are tightly linked to the characteristic spatial distribution of visual objects. Importantly, since these studies typically use meaningless checkerboard stimuli to map RF properties, their findings demonstrate that visual field biases exhibited by category-selective regions are evident even in the absence of any categorical processing demands. That is, when no categorical information is present in the stimulus, the field of view of a region cannot be adjusted based on content-specific feedback processes. These population RF mapping studies therefore corroborate the notion that our extensive experience with real-world environments influences neural tuning independently from top-down feedback (Box 1).

The conjoint tuning to object category and visual field location yields measurable benefits in perceptual performance: across individuals, RF sizes in face-selective and word-selective cortex, respectively, predict face recognition performance [33] and reading speed [24]. At a finer-grained level, the characteristic spatial coverage of object-selective neurons may predispose the enhanced representation of typically positioned objects even within a category [12–14].

Which level of representation is enhanced when objects are positioned in their typical real-world locations? The fact that the effects of typical positioning are observed in high-level visual cortex suggests that they are not caused by visual field biases in low-level feature processing. However, these regions represent a multitude of object properties ranging from object-associated mid-level attributes (e.g., the characteristic shape or texture of an object) to categorical object content. Because these organizations are spatially entwined [34,35], it is currently unclear whether the preferential processing of typically positioned objects reflects differences in object-level representations, or in the representation of object-associated mid-level features, or both.

To summarize, recent findings provide convergent evidence that the cortical object-processing architecture is tailored to the spatial distribution of objects in the real world. Consequently, object perception varies systematically across the visual field, with more efficient processing for individual objects appearing in their typical absolute locations in the world.

## Adaptations to Relative Locations in Multiobject Processing

**Figure 2:**
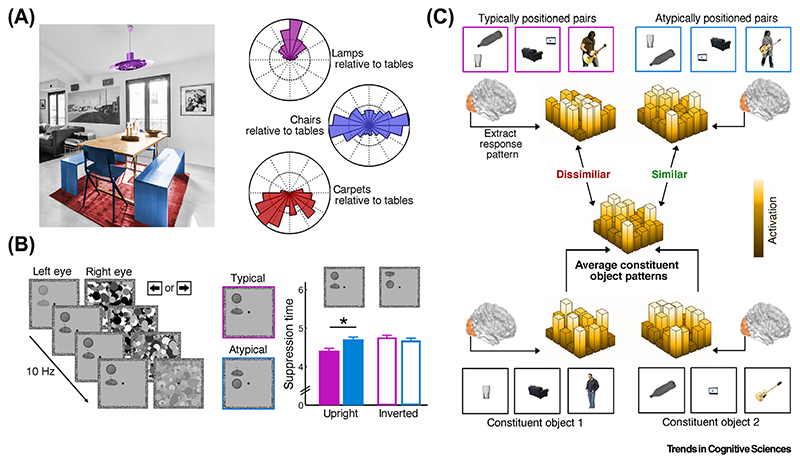
Neural Adaptations to Typical Relative Object Locations. (A) In addition to regularities in absolute object positions, the structure of natural scenes also yields regularities in the locations of objects relative to each other. For example, dining tables typically appear beneath lamps, above carpets, and surrounded by chairs. Polar plots illustrate the position of lamps, carpets, and chairs, all relative to tables, across photographs from the LabelMe toolbox [129]. (B) When multiple objects are positioned in their typical relative locations, they are preferentially detected under continuous flash suppression (CFS). Similarly to the effects of absolute positioning, regularities in relative positions thus grant benefits in basic perceptual processing. Notably, because stimulus inversion abolishes these effects, they are not explicable by low-level factors. Data reproduced from both experiments in [36]. (C) At a neural level, the advantages for typically positioned multiobject arrangements may arise from aggregating individual objects into group representations, as indicated by fMRI studies comparing multivoxel response patterns evoked by multiobject displays and their constituent individual objects. These studies show that multiobject response patterns are well predicted by an average of the individual-object response patterns when the objects are atypically positioned, indicating independent processing. Crucially, when the objects are typically positioned, the multiobject pattern is not as accurately predicted, indicating additional integrative neural processes. Such results have been demonstrated for grouping based on action relationships (e.g., a bottle pouring water into a glass [59]), real-world co-occurrence (e.g., a sofa facing a TV [61]), and person-object interactions (e.g., a person playing a guitar [60]).

Natural environments are inherently structured not only in terms of the absolute locations of objects within the environment, but also in terms of the relative positioning of objects with respect to each other. For example, objects in a dining room typically appear in specific relative locations (e.g., chairs typically surround a table, with a lamp above and a carpet below) (Figure 2A). Such statistical regularities in the relative positions of objects influence object processing in systematic ways, in the same way as regularities in the absolute locations of objects influence such processing.

Exactly as the typical absolute positioning of objects impacts on basic levels of perceptual processing, so too does their typical relative positioning: under CFS, observers detect groups of typically arranged objects (e.g., a lamp above a table) faster than groups of atypically arranged objects (e.g., a lamp below a table) [36] (Figure 2B), even when the task does not require explicit object recognition. Importantly, a control experiment dissociated the relative-position benefit from the absolute positions of the constituent objects [36], showing that both typical relative positioning and typical absolute positions facilitate object detection under CFS.

Beyond basic detection, perceptual benefits associated with typical relative positioning are found in explicit identification and recognition tasks, where typically positioned groups of objects [37–41] and interacting groups of people [42–44] are easier to perceive. Typical relative positioning also facilitates memory: perceptual detail of multiobject and multiperson displays is more accurately maintained in visual memory when the display is arranged in accordance with real-world positional regularities [44–50], suggesting that typical relative positioning facilitates the representation of multiobject information in both perceptual and cognitive systems.

Why are typically positioned object arrangements represented more efficiently? One possibility is that multiple objects arranged in their typical relative positions are represented as a group rather than as multiple individual objects, thereby reducing the descriptive complexity of multiobject representations. For instance, a table flanked by chairs with a lamp above and carpet below may be represented as a single ‘dining group’, rather than as multiple individual objects. This idea is reminiscent of the study of grouping in low-level vision (Box 2), where the emergence of perceptual **Gestalt** has been associated with the grouping of different pieces of visual information. Interpreted in a similar way, the studies reviewed above could reflect the grouping of objects when they appear in accordance with real-world structure.

This assertion has been tested at the neural level, where grouping is mediated by **integrative processing** of objects. This would lead to enhanced activations in visual cortex for objects in typical versus atypical relative positions. Such enhanced activity has indeed been observed for objects that co-occur in real-world scenes [51], objects that form relationships based on motor actions [52–54], faces on top of bodies [55,56], and even for multiple people engaged in social interactions [57,58].

Although these studies are in line with integrative processing, increases in overall activity may partly reflect other factors such as greater attentional engagement with the typically positioned objects. Therefore, as an alternative measure of integrative processing, recent studies have investigated how relative object positioning affects the similarity of multivoxel response patterns in the absence of overall activity differences [59–61] (Figure 2C). These studies were inspired by the integration of simple visual features based on Gestalt laws, where ‘the whole is something else than the sum of its parts’ [62]. The use of **multivoxel combination analysis [63]** allows testing of whether a similar principle underlies the representation of multiple objects in visual cortex. When multiple objects are processed independently, a linear combination of the individual-object patterns (e.g., the mean) accurately predicts the multiobject pattern [64–66]. However, when multiple objects form a coherent group, the multiobject pattern (the ‘whole’) is relatively dissimilar to the linear combination of individualobject patterns (the ‘parts’).

In one such study [61], pairs of objects were positioned either as they would typically appear in real-world scenes (e.g., a sofa facing a TV) or were atypically arranged (e.g., a sofa facing away from a TV). Response patterns to the object pairs were then modeled as the mean of response patterns evoked by the constituent objects individually (e.g., sofa and TV, each in isolation). Multiobject patterns in object-selective LOC were less accurately modeled by the individualobject patterns when the objects adhered to their typical real-world positioning, providing evidence for integrative processing based on typical relative object position. Evidence for neural integration of typically positioned arrangements has also been found for other types of high-level content: for meaningful human-object interactions (e.g., a person playing a guitar), individual-object patterns did not accurately explain response patterns in the posterior superior temporal sulcus [60], and, for action relationships between objects, combination weights in LOC were altered when objects were positioned correctly for action (e.g., a bottle pouring water into a glass) [59].

At a mechanistic level, these effects are parsimoniously explained by the involvement of additional neural assemblies that exclusively represent typically positioned object groups. Through extensive exposure to concurrent objects appearing in typical relative locations, specialized neural assemblies may become tuned to the concerted presence of these objects [67–69]: consequently, these neural assemblies start to respond exclusively to the presence of the objects in their typical relative locations. These additional responses would not only enhance activations to typically positioned object groups but also distort the multivoxel response patterns they evoke. As a complementary mechanism, multiple objects may be bound by connectivity between the individual-object representations, establishing enhanced functional coupling between these representations (e.g., through neural synchrony [70,71]). Although there is evidence for such increased functional coupling between representations of features belonging to the same object [72,73], future studies need to test whether representations of multiple distinct objects can be bound in similar ways.

Another open question concerns the level of representation at which object information is grouped. Given that the effects of typical relative positions emerge in anterior parts of LOC [61], it is possible that grouping reflects an integration of high-level object representations. Alternatively, it could also reflect the integration of object-associated mid-level features, such as characteristic object shape. For example, along the vertical axis, large square-shaped objects are more often found below than above smaller objects of various forms. However, previous studies suggest that grouping does not exclusively rest on the combination of such characteristic mid-level features: grouping effects are stronger when typically positioned objects are strongly semantically related (e.g., lamp above table, mirror above sink) compared to when they are less related (e.g., mirror above table, lamp above sink) [48,74]. Similarly, grouping is reduced for people performing actions directed towards unrelated objects rather than to meaningful recipients [42].

Together, these findings suggest that the perceptual benefits observed for typically positioned multiobject arrangements reflect the grouping of multiple individual object representations. This grouping mechanism may be of particular relevance in the context of complex real-world scenes, wherein individual objects often form meaningful arrangements, such that integrating their individual representations into a higher-order group representation may serve to effectively simplify scene analysis.

## Adaptations to Real-World Structure Reduce Multiobject Competition

The findings reviewed here collectively suggest that sensitivities to real-world spatial structure are ubiquitous in high-level vision: we can observe them in the neural representations of both individual objects and multiobject arrangements, as well as across a diverse range of high-level visual stimuli. We posit here that a common purpose underlies these various adaptations to real-world structure: namely, the optimal use of limited cortical resources. In the following we first reflect on the nature of cortical resource limitations, and then outline how adaptations to both typical absolute locations and typical relative locations allow us to efficiently represent objects in the context of these limitations.

A unifying commonality across perceptual and cognitive systems is their restricted capacity to process multiple entities simultaneously [75,76]. Indeed, it is well established in the low-level visual processing literature that perceptual performance is drastically compromised when multiple items compete for simultaneous representation (e.g., when searching in visual clutter [77,78]). Such difficulties in perceiving multiple objects simultaneously are tightly linked to competition effects at the neural level [79-81]: when stimuli directly compete for overlapping processing resources (e.g., when multiple objects fall within the RF of a given neuron), the response to each individual stimulus is reduced - a detrimental effect that increases in proportion with processing overlap.

In the context of real-world vision, the representational deficit imparted by interobject competition yields pessimistic predictions: most natural scenes comprise a large number of objects [82], many of which share visual and/or conceptual properties and therefore compete for the same neural processing resources. Recognizing individual objects in the face of such intense competition should be extremely challenging for the brain - but our experience in natural vision is exactly the opposite. We seem to effortlessly recognize objects even when these objects are embedded in highly complex scenes [83,84]. We suggest here that this striking discrepancy is partially accounted for by perceptual adaptations to real-world structure. Specifically, we propose that the visual system exploits the systematic spatial distribution of objects in its environment to reduce the degree to which these individual objects compete for neural processing resources. Adaptations to both the typical absolute location (in space) of an object and its typical relative location (to other objects) contribute towards this goal of reducing interobject competition. We argue that (i) typical absolute locations reduce competition through sharpened and more efficient representations of individual objects, and (ii) typical relative locations reduce competition by integrating multiple objects into group representations.

First, adaptations to the typical absolute locations of objects can reduce interobject competition by increasing the precision of neural representations. Classical theories of object recognition [85, 86] typically assume that high-level vision converges towards invariant object representations which are tolerant to variation in the location of an object. Consistent with this notion, receptive fields of individual neurons in high-level visual cortex span larger areas of visual space [87]. However, at a population level, object-selective regions of the ventral visual cortex nonetheless retain relatively precise information about object location [88–90], suggesting that object recognition may not be ultimately position-invariant [91]. Indeed, to efficiently process objects without losing information about their visual field locations, an ideal visual system would be able to support a precise representation for any given object at any possible location. In light of its limited processing resources, however, our visual system must make compromises in representational precision: because lamps reliably appear in the upper visual field, supporting a precise representation of lamps in this part of the visual field is a reasonable investment, whereas maintaining an equally precise representation in other parts of the visual field is not. As outlined above, there is mounting evidence in the neuroimaging literature for such location-governed tradeoffs in representational precision [12–14,20].

**Figure 3:**
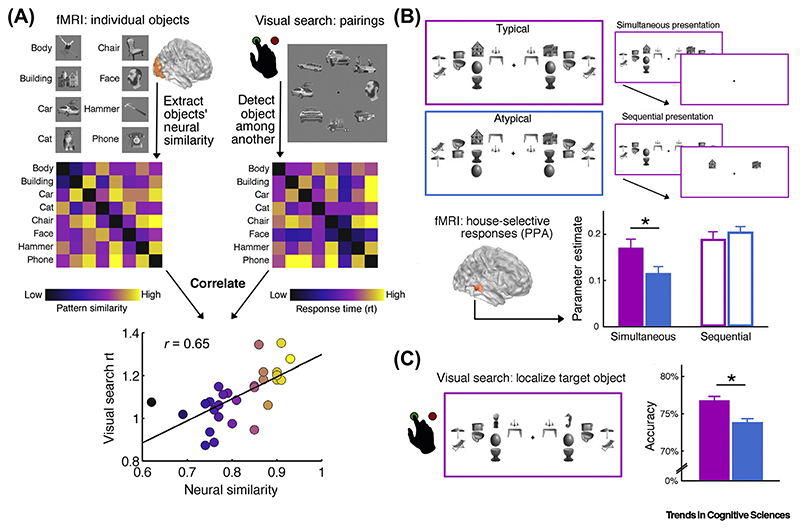
Adaptations to Positional Object Regularities Reduce Multiobject Competition. (A) Representational separation reduces multiobject competition. At a categorical level, visual search performance is predicted by the overlap in cortical processing of the target and distracters. For example, where there is high overlap in the neural representations of cars and telephones (i.e., they evoke similar fMRI response patterns), there is comparatively less representational overlap between cars and faces. Consequently, finding a phone among cars is comparatively slower than finding a face among cars. At a more fine-grained level, representing individual objects via distinct categorically and spatially tuned neural channels may also serve to reduce response overlap and thereby facilitate multiobject representation. Data reproduced from [95]; in the original study, stimuli were additionally matched for spatial frequency content. (B) Reducing multiobject competition by grouping objects in typical relative positions. In fMRI, unrelated objects (houses) evoke stronger selective cortical responses when surrounding object pairs conform to their typical relative positions (e.g., mirror above sink). This processing benefit for typically positioned object pairs is eliminated by temporally separating the houses and object pairs (i.e., sequential presentation), suggesting that the effect reflects reduced cortical competition between concurrent stimuli - even though neither the houses nor the object pairs were task-relevant. Abbreviation: PPA, parahippocampal place area. (C) Complementary effects are found in visual search among similar displays: consistent with the reduction of competition at a neural level, participants localize unrelated target items more accurately when distracter pairs are positioned typically rather than atypically. These results show that grouping based on typical relative positions reduces multiobject competition, thereby simplifying the perception of cluttered scenes. Data reproduced from [74].

The preferential processing of particular objects by separate, spatially tuned neural populations is further apparent at the level of visual categories. In high-level visual cortex, spatially distinct regions that process information for various visual categories (e.g., scenes, faces, or words) can be differentiated both in terms of how they sample visual space [24,31] and in terms of their connections with retinotopic mechanisms at lower levels of the visual hierarchy [92]. This separation of category processing into discrete categorically and spatially tuned channels can be linked to efficiency in multiobject processing [93–95]. For example, visual search performance is determined by the cortical similarity between the target and distracter categories [95] (Figure 3A): detecting a phone among cars is difficult (because their neural representations overlap substantially and thus compete substantially), whereas detecting a face among cars is easy (because their neural representations overlap less and thus compete less). This link between processing overlap and perceptual efficiency suggests that more precise and less overlapping representations of individual objects appearing in typical locations also exhibit less competition. This mechanism may be highly beneficial in cluttered scenes that contain multiple objects, many of which appear in typical locations within the scene.

Second, adaptations to the typical relative locations of objects may reduce interobject competition by effectively reducing the number of objects competing for resources. Where a visual system with infinite processing resources could afford to process all objects in parallel (e.g., a table and a lamp), the biological constraints on the human visual brain are such that grouping objects (e.g., into a lamp-and-table group) becomes an efficient way to reduce the number of individual items competing for representation. Although the representations of object groups are still subject to resource-capacity limitations, competing for resources at a group level results in fewer representations in direct competition with one another, and consequently this competition is less detrimental than when representing the objects individually.

The notion that integrating information carried by co-occurring objects reduces interobject competition is borne out by neuroimaging work showing that object-category responses (e.g., neural activity in parahippocampal cortex evoked by a house) are stronger when concurrently presented competitor objects can be grouped based on their typical positioning (e.g., a lamp above a table, a mirror above a sink) than when they cannot be grouped based on their positioning (e.g., a table above a lamp, a sink above a mirror) [74] (Figure 3B). Notably, in this experiment neither the houses nor the competing objects were behaviorally relevant, suggesting that multiobject grouping occurs automatically during perceptual analysis. The enhanced processing of stimuli embedded in typically positioned object arrays also plays out in human behavior: in a complementary visual search experiment (Figure 3C), participants could detect targets more accurately when distracters could be grouped based on real-world regularities [74]. Together, these results suggest that interobject grouping reduces multiobject competition, and thereby simplifies the representation of complex scenes.

In sum, adaptations to real-world structure can reduce neural competition between objects in multiple, complementary ways: (i) adaptations to typical absolute locations reduce processing overlap between representations precisely tuned for particular objects appearing at particular locations, and (ii) adaptations to typical relative locations allow multiobject grouping, thereby reducing the number of objects competing for processing. Together, these adaptations simplify the neural code for scene analysis. The resulting simplification of scene representation contributes to the efficiency of human performance in naturalistic tasks, such as visual search in scenes [9,11,83,84] or scene memory [6,96].

## Concluding Remarks and Future Directions

The current review underscores the fundamental and intrinsic link between the structure of natural environments and our visual perception of the world. The adaptations reviewed here support effective object processing in the real world: by capitalizing on positional regularities, the visual brain is able to optimally represent complex multiobject scenes. To conclude, we briefly revisit four key insights of our review and delineate how these insights inspire future research in object vision and beyond (see Outstanding Questions).

First, adaptations to real-world structure play a key role in the perception and representation of various types of high-level content, including diverse everyday objects (e.g., furniture, tools, landmarks), human beings (e.g., faces, bodies, and their component parts), social and functional action relationships (e.g., between people and/or objects), and written text. This not only shows that high-level vision is inseparably linked to real-world structure but also highlights that positional regularities play a key role in many everyday tasks, from action understanding to reading.

Second, adaptations to real-world structure arise in both cognitive and perceptual systems. Most interestingly, they not only influence high-level processes such as recognition and working memory, but also operate at the very early stages of visual processing, even determining how quickly we detect an object in the first place. This shows that real-world positional regularities exert a more fundamental influence than was previously thought: not only do they equip humans with cognitive strategies to explore the world in smart ways, they also support the efficient perceptual parsing of natural information.

Third, the study of positional regularities in high-level vision could advance current efforts in modeling the human visual system. The far-reaching impact of real-world structure suggests that object vision cannot be fully understood without taking real-world structure into account. The recent insights thus urge a consideration of positional regularities in neural models of object processing. Interestingly, explicitly considering real-world structure may not only help to understand the biological brain but also fuel developments in computer vision (Box 3).

Finally, the importance of real-world structure supports neurocognitive research that pushes towards more naturalistic approaches to vision: only by studying vision under conditions that more closely mimic the properties of real-world environments will we come closer to understanding how we efficiently select, recognize, and ultimately extract meaning from a complex visual world.

## Figures and Tables

**Figure I:**
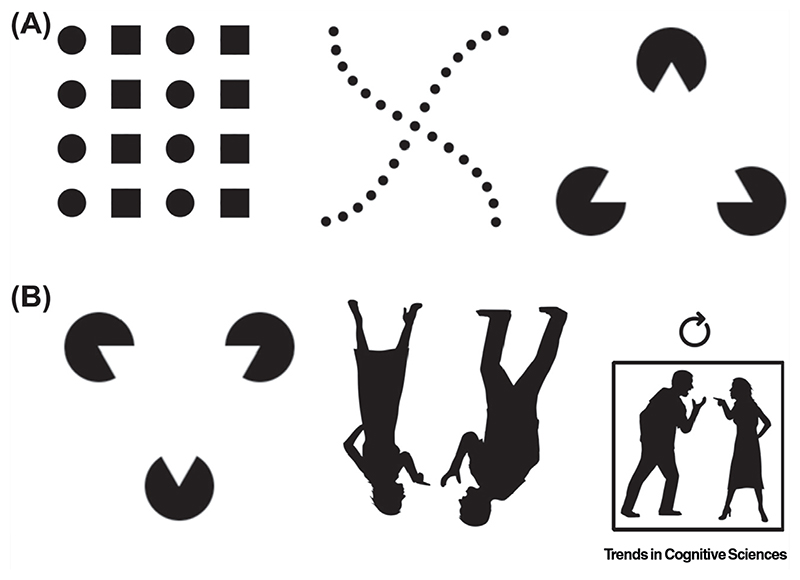
Regularities in Low-Level Vision. (A) Examples of Gestalt formation through low-level grouping based on (left to right) similarity, good continuation, and illusory contour formation. (B) Regularities in low-level vision (e.g., grouping by contour formation) are unaffected by inversion. By contrast, high-level regularities (e.g., the grouping of multiple social agents [43]) are disrupted upon inversion.

## Box 1 Origins of Cortical Adaptations to Real-World Structure

When and how do cortical adaptations to real-world structure emerge? One possibility is that these adaptations reflect experience-based changes in neural tuning. This view is supported by perceptual learning studies that show that cortical tuning to specific low-level features and their conjunctions is enhanced in a spatially specific way [99]. In line with this idea, recent fMRI results show that RF biases in face- and word-selective cortex are shaped across development [100,101], suggesting a key role for visual experience in the formation of RF properties in these regions. Alternatively, visual field biases could be an inherent property of the cortical architecture, and thus be in place even before visual experience plays out [30,102,103]. On this possibility, suitable neural assemblies are subsequently ‘conquered’ by stimuli that require their specific tuning properties. This view is supported by the observation that category-selective regions are characterized by unique structural fingerprints such as their cytoarchitecture [104,105] and connectivity with other brain regions [106,107]. Interestingly, the connectivity patterns of visual regions can be in place before experience can sculpt their functional profile [108], and are remarkably similar in the absence of visual experience [109]. In the end, both mechanisms may be at work, with pre-existing and rigid architectural properties being refined by moderate changes in cortical tuning in response to visual experience [110].

## Box 2 Positional Regularities in Low- and High-Level Vision

Natural environments are structured not only in terms of high-level object content but also with respect to low-level visual attributes [111,112]. A prime example in this regard is the emergence of perceptual Gestalt from the grouping of multiple simple stimuli (Figure IA). Such low-level grouping modulates neural responses both in early visual cortex and in higher-level shape-selective regions [113–115]. As for real-world object regularities, multivoxel combination analysis suggests that these activation differences reflect the integration of stimulus components into group representations [116]. Moreover, this neural integration confers a behavioral advantage in capacity-limited tasks such as visual search [117–119] or working memory [120–122]. Such analogies raise the question of the extent to which the underlying cortical adaptations to low- and high-level regularities differ. First, the two effects may be situated on different levels of the processing hierarchy because recent evidence indicates that multiobject grouping effects arise later in the hierarchy than basic object sensitivity [61]. Second, the two types of regularities may arise from different mechanisms because low- and high-level effects can be dissociated using inversion effects [36,43,48,74] (Figure IB). Despite these dissociations, the cortical tuning to low-and high-level regularities may be based on similar principles, such as the grouping of multiple elements to pass capacity bottlenecks. Future research therefore needs to explicitly juxtapose regularities on different levels to extract such common principles in their implementation.

## Box 3 Real-World Structure for Computer Vision

A promising avenue in which the study of adaptations to real-world regularities might fuel future developments concerns computer vision. In recent years, deep neural network (DNN) models have approached human performance in a variety of visual tasks [123,124]. These DNNs are typically trained on large and diverse sets of natural images, which inevitably exposes them to the inherent positional structures of scenes - but DNN training can also be enhanced by explicitly emphasizing real-world structure. We highlight here three ways in which adding explicit information about real-world structure could improve DNN models of vision. First, augmenting DNN training procedures with explicit information about real-world regularities (for example using human-derived object expectations [125]) could increase DNN performance levels in tasks that strongly rely on recurring spatial regularities (e.g., assisted driving). Second, in addition to improving their task performance, augmenting DNNs with real-world structure could also make their performance more human-like. Creating computational models that accurately mirror human vision would be tremendously helpful for predicting human decisions in naturalistic tasks. For example, humans are sometimes fooled by their knowledge about real-world structure, such that, in contrast to DNNs, they miss targets that do not align with their expectations (e.g., when targets are wrongly scaled given the scene context [126]). Constructing computer models that more strongly take scene structure into account, we would be able to foresee different types of errors in real-life situations and prevent them by warning humans accordingly (e.g., by warning drivers about otherwise missed hazards). Third, although DNNs have recently become the state-of-the-art model for accurately predicting visual brain activations [127,128], by no means do they explain object processing in full. Given the importance of real-world structure for human vision, enriching DNNs with positional regularity information has the potential to further improve the concordance between DNN models and the human brain. In turn, by understanding how DNNs change when explicit information about positional regularities is included during training, we can make predictions about the mechanistic implementation of regularity information in the brain.

## Highlights

The characteristic spatial distribution of individual objects impacts profoundly on object processing in the human brain. Neural representations are sharper and perceptual sensitivity is increased when objects appear at retinal locations corresponding to their typical location in the world (e.g., an airplane in the upper visual field).

The characteristic positioning of objects relative to one another also systematically influences object processing. When multiobject displays are arranged in their typical relative positions (e.g., a lamp above a table), the visual system represents them as groups, allowing objects to be more easily detected, recognized, and memorized.

Neural adaptations to natural scene structure enable the visual brain to optimally represent the large number of objects contained in real-world environments, thereby simplifying the neural code for scene analysis.

## Glossary

### Abbreviations

Continuous flash suppression (CFS): a psychophysical method to study access to visual awareness. During CFS, a static stimulus is shown to one eye (e.g., via anaglyph glasses) while a dynamic contrast-rich mask is repeatedly flashed to the other eye (e.g., 10 masks per s), rendering the static stimulus invisible or ‘suppressed’ for a sustained period (e.g., a few seconds). The elapsed time before an observer successfully detects or localizes the stimulus is often taken as a measure of the ability of the stimulus to access visual awareness [16,17].
Gestalt: a meaningful whole emerging from the arrangements of multiple and simpler components. Gestalt principles refer to the laws that govern the integration of simple visual elements into a coherent percept.
Integrative processing: processing that combines multiple representations into composite representations.
Multivariate pattern analysis (MVPA): an analysis technique that capitalizes on pattern information in neural recordings. Whereas classical univariate analyses compare mean activations across conditions, multivariate analyses compare distributed activations. Multivariate analysis approaches are widely used to study cortical representations using fMRI [97] and magnetoencephalography/ electroencephalography (M/EEG) [98].
Multivoxel combination analysis: a variant of MVPA for fMRI, where response patterns evoked by individual objects are used to model response patterns evoked by multiple objects. If a linear combination of the individual response patterns (e.g., their mean) is an accurate approximation of the group pattern, the objects are processed independently. Conversely, if the group pattern is less-accurately approximated by the linear combination of individual response patterns, additional integrative processes are involved [63].
Population receptive-field mapping: a method for estimating the RF properties of neural assemblies based on fMRI responses. Typically, high-contrast stimuli are moved across the visual field in a systematic way, and the resulting data are used to model RF positions and sizes across visual space (reviewed in [23]).
Receptive field (RF): the region of (visual) space where stimulation changes the firing behavior of a neuron.

## Outstanding Questions

If the various adaptations to real-world positional regularities serve a common goal of simplifying scene analysis, can we also uncover common principles in their implementation? For example, is the grouping of typically co-occurring objects (e.g., a lamp above a table) qualitatively similar to the mechanism which facilitates integrating information across social agents (e.g., multiple people involved in an argument)?

Conversely, does the sensitivity of the visual system to positional regularities differ across domains? If so, how are such differences related to the different nature of regularities forming on the basis of physical constraints (e.g., object arrangements obeying the laws of physics), social situations (e.g., multiple people interacting), and societal conventions (e.g., the composition of written text in a specific language)?

Thus far, sensitivities to real-world positional regularities have typically been investigated using 2D static images. How do these adaptations manifest under more naturalistic viewing conditions, where specific objects appear at particular depths and exhibit characteristic movement patterns across time?

Does real-world structure shape perceptual architecture in modalities other than vision? Further, can future studies on real-world regularities facilitate our understanding of multisensory integration in naturalistic scenarios?

How flexible are adaptations to real-world structure? How fast can observers adapt to novel environments whose structure differs radically from the one they typically navigate in?

Can we use insights regarding the importance of scene structure to improve the design of our everyday environments? For example, can studies on real-world regularities inspire smart and easy-to-perceive designs in architecture, civil engineering, or art?
